# Has the COVID-19 Pandemic Accelerated the Future of Work or Changed Its Course? Implications for Research and Practice

**DOI:** 10.3390/ijerph181910199

**Published:** 2021-09-28

**Authors:** Matthew A. Ng, Anthony Naranjo, Ann E. Schlotzhauer, Mindy K. Shoss, Nika Kartvelishvili, Matthew Bartek, Kenneth Ingraham, Alexis Rodriguez, Sara Kira Schneider, Lauren Silverlieb-Seltzer, Carolina Silva

**Affiliations:** 1 Psychology Department, University of Central Florida, Orlando, FL 32816, USA; anaranj2@knights.ucf.edu (A.N.); aeschlotz@knights.ucf.edu (A.E.S.); mindy.shoss@ucf.edu (M.K.S.); nikokartvel@gmail.com (N.K.); mabartek14@knights.ucf.edu (M.B.); kenneth.ingraham@knights.ucf.edu (K.I.); acr15b@knights.ucf.edu (A.R.); kiraschneider@knights.ucf.edu (S.K.S.); lauren.seltzer@knights.ucf.edu (L.S.-S.); carolinapsyc@knights.ucf.edu (C.S.); 2 Peter Faber Business School, Australian Catholic University, Melbourne 3065, Australia

**Keywords:** COVID-19 pandemic, future of work, work arrangements, compensation and benefits, worker well-being, occupational safety and health, work organization-related chronic health conditions, including substance use disorders, work-family conflict, trends

## Abstract

The COVID-19 pandemic is a unique transboundary crisis which has disrupted people’s way of life more dramatically than any event in generations. Given the ambiguity surrounding the end of the COVID-19 pandemic and its enduring negative effects, it is important to understand how this has affected important future of work trends. The aim of the current paper is to assess the impact of the COVID-19 pandemic on commonly discussed future of work trends relevant to occupational safety and health priority areas. These topics include work arrangements, compensation and benefits, and the organization of work. For each topic, we assess trends leading up to the COVID-19 pandemic, discuss the impact of the pandemic on these trends, and conclude with implications for research and practice. Overall, the pandemic appears to have both accelerated and disrupted various trends associated with future of work topic areas. These effects are discussed in terms of implications for both policymakers and organizations.

## 1. Introduction

Landmark events are those events that are said to change history. The COVID-19 pandemic (pandemic), already in its second year, will certainly stand as one such event. As of the writing of this paper, over 4.5 million people worldwide have died from COVID-19, and a new variant is rapidly surging in the United States. The onset of the pandemic brought rapid shifts to remote work, hundreds of thousands of job losses due to business shutdowns and closures, school closures, and new work hazards; these challenges have persisted throughout the pandemic. The pandemic has been labeled a transboundary crisis—a crisis that impacts all elements of a social system [1]. With its major impacts on work and work-related well-being, it has been described as “the most widespread and profound occupational health crisis in modern times” [2].

There has been much speculation about how the pandemic has changed the future of work. We leverage available data to examine how the pandemic is shifting trends in the United States surrounding work arrangements; policies, compensation, and benefits; and the organization of work. We identified these themes as major disruptions sparked by the pandemic that are associated with future of work topics and have the potential to impact occupational safety and health priorities related to worker well-being. Moreover, these are topics for which empirical data exists to facilitate the comparison of trends. Thus, these topics provide a useful starting point to consider how the pandemic is shaping the future of work from an occupational safety and health perspective, and to generate ensuing implications for occupational safety and health research and practice. Although many rich discussions could be had about the pandemic’s impact across countries, we restrict our scope to the United States given the focus of this special issue.

In each of the sections that follow, we incorporate previous research findings and available data to synthesize trends both before and during the pandemic for each topic, and then generate implications for research and practice. Across the sections, we address cross-cutting issues including workforce demographics and inequities. We conclude this paper by offering several overarching insights about the ways that research and practice priorities related to the future of work may need to change due to the pandemic.

## 2. Work Arrangements

Most prognostications of the future of work focus on questions about whether work will continue to exist in the manner it has in the past. These discussions generally involve the disruptive nature of technology in shaping where work is done (e.g., in the office versus virtually), the nature of the employment relationship (e.g., through gig work and non-standard employment relationships), and whether work will even continue to exist for large portions of the population [3,4,5,6]. Such questions about work arrangements are highlighted in occupational safety and health and future of work frameworks, which emphasize the interconnection between work arrangements and physical, psychological, social, and economic impacts of work [7].

Work arrangements, or “arrangements between those who perform work and those who provide jobs”, are commonly discussed as standard and non-standard [8] (p. 1). Standard work arrangements refer to more traditional employment, wherein individuals work long-term, full-time jobs for the same employer, typically with some ability to advance their career over time [9]. Work arrangements that vary in one or more ways from this pattern are considered non-standard, or flexible, work arrangements [9,10]. This can include independent contract work, remote work, telework, temporary help agency work, freelance work, on-demand or gig work, part-time work, and more [8,9,10]. This section first focuses on two types of work arrangements highly impacted by the pandemic, remote work arrangements and gig work, before pivoting to consider the displacement of work caused by the pandemic.

### 2.1. Remote Work

#### 2.1.1. Pre-Pandemic Trends

As technology supporting remote work has continued to advance, a shared physical workspace has grown less necessary in many industries, especially for those involved in knowledge work. Remote work appeals to many workers due to its flexibility and the savings associated with not commuting [11]. From the organization’s perspective, cutting the costs associated with renting and maintaining a physical space can translate to significant savings [12]. Simultaneously, flexible work arrangements open organizations to a wider talent pool, allowing them to hire the best people for the job, regardless of physical location; this has the added benefit of increasing the diversity within workgroups, promoting creative problem solving and increasing access to jobs [13,14]. Despite these advantages, remote work was far from the norm leading up to the pandemic. In fact, in 2019, the USA Bureau of Labor Statistics (BLS) reported that 29% of wage and salary workers could work at home and 25% had opted to do so at some point [15]. However, only 15% of employees reported working full days exclusively from home [15]. These figures had advanced little over the preceding decade, suggesting rates of remote work had been largely stagnant despite it being hailed as a hallmark of a technology-mediated future of work [16].

Several circumstances may have contributed to remote work’s limited popularity. First, working from home is not a realistic option in many industries. In the aforementioned BLS data [15], the percentage of workers who could work from home in the financial (57%) or information (53%) sectors dwarfed the percentages who reported the same opportunity in leisure and hospitality (9%) or agriculture (11%). Even when remote work is an option, opinions about its efficacy are mixed. Communication that occurs between individuals in the same physical environment allows for greater richness, creating potentially costly barriers when digital communication is poorly leveraged [17,18]. Research suggests knowledge sharing amongst coworkers is negatively affected by both spatial and temporal separation [19]. Remote workers may also experience feelings of isolation, which can lead to stress and decreases in affective commitment [20,21]. Further, physical presence in the office is often interpreted as commitment or “willingness to perform” [22] (p. 13); as a result, remote work may have negative effects on career outcomes [23]. Importantly, a lack of physical presence in the office may not affect all employees equally. Indeed, remote working women, and especially mothers, have reported decreased visibility and concerns about the impact on their career progression [24]. Finally, if poorly managed, employees engaging in remote teamwork may experience more intra-team conflict and struggle to maintain trust, which has implications for well-being [14,25].

#### 2.1.2. Pandemic Trends

Perhaps the most visible effect the pandemic has had on the average office has been emptying it. Particularly at the outset of the pandemic, massive sectors of employees transitioned from primarily working onsite to working remotely from home [26,27]. Compared to roughly 15% of employees occasionally working full days at home prior to the pandemic [15], BLS estimated 35% of the workforce worked from home due to the pandemic at least once in May 2020 [26]. Of note, this data captures those forced to work from home due to the pandemic; adding those who already worked full-time from home prior to the pandemic would inflate the figure even further. This disruption led many in the popular press to speculate whether the pandemic has served as a catalyst for a long-term exodus from the traditional working situation [28,29].

As governmental restrictions are lifted and companies relax their policies, fewer individuals are being forced to work from home. By the end of 2020, those forced to work from home as a result of the pandemic had fallen to 24% [26]. By July of 2021, only 13% reported that they had been forced to work from home at some point in the past four weeks due to the pandemic [26]. Even with this steady decline from remote work’s peak in spring 2020, there are still more Americans working from home than before the pandemic [26]. This raises the question of how many employees will continue to work from home after it is completely safe to return to the office. The answer will likely depend on a mixture of organizational decision-making and individual workers’ preferences.

Attitudinal surveys provide a useful preview of worker preferences. Between October 2020 and April 2021, Gallup polled white-collar workers, who are typically more able to work remotely, on their preferences [30]. Of those white-collar workers currently working from home, 71% wanted to continue working from home [30]. While 15% of those polled preferred remote work due to concerns about COVID-19, the remaining 56% reported they wanted to continue remote work as a matter of personal preference [30]. Conversely, only 29% reported wanting to return to the office [30]. Similarly, a poll by the Harvard Business School found 81% of professionals who had been working remotely from March 2020 to March 2021 either did not wish to return to the office or wanted to telework via a hybrid schedule (e.g., working 2–3 days at home per week) [31]. Notably, many white-collar industries (e.g., information, professional and business services) are predominantly composed of white and male employees [32], introducing concerns about the availability of remote work opportunities for traditionally disadvantaged groups.

As individual employees’ opinions have shifted, a number of high-profile companies have announced decisions to permanently move employees to hybrid schedule telework or full-time remote work [33,34]. In fact, the Society for Human Resource Management reported that 18% of organizations polled do not plan to bring all employees back to the worksite even when it is safe to do so [35]. Considering these factors jointly, there is good reason to expect a long-lasting shift in remote and telework’s popularity, particularly in well-suited industries like professional business and services [36].

### 2.2. Gig Work

#### 2.2.1. Pre-Pandemic Trends

Gig work can be understood as “a single project or task for which a worker is hired, often through a digital marketplace, to work on demand” [37]. Contingent workers, who do not have an expectation of long-term employment, are subsumed under this category [38]. Varying definitions mean that gig workers “are not easily identified in surveys of employment and earnings” and estimates of gig work’s popularity vary [37,39,40]. For example, the most recent pre-pandemic BLS data provided three estimates of the percentage of people employed in contingent work ranging from 1–4% of those employed [38]. In addition to contingent workers, BLS reported that approximately 10% of workers were engaged in alternative work arrangements, including independent contractors, on-call workers, temporary help agency workers, and workers provided by contract firms [38].

Despite forecasts of gig work’s prevalence skyrocketing [41,42], the available data represents very little change from previous BLS estimates in 2005 and 1995 [43]. Certain aspects of the work may account for this lack of growth. For example, gig work’s inconsistency and lack of benefits are concerns for many employed in this sector [37]. Further, research has indicated that those in non-standard work arrangements are generally exposed to more occupational safety and health risks and report greater musculoskeletal and mental symptoms than those in standard work arrangements [44]. Nonetheless, a Gallup poll found 64% of gig workers reported doing their preferred type of work [45]. This suggests that, at least prior to the pandemic, many gig workers willingly accepted the risks of a non-traditional work arrangement.

#### 2.2.2. Pandemic Trends

The pandemic’s effects on gig work are less straightforward than its impact on remote work. Certain segments of the gig economy have thrived. For example, Instacart added 300,000 new shoppers between March and July 2020 to address growing demand; meanwhile, Upwork, which connects freelancers with employers, reported a 50% increase in signups from both workers and employers [46]. At the same time, consumer demand for rideshare companies (e.g., Uber and Lyft) fell by 75–80% in April 2020, compared to April 2019 [46]. Similarly, Airbnb suffered a 70% drop in bookings at the outset of the pandemic [47]. However, as the pandemic has progressed, demand has shifted. Airbnb, for example, has recovered, benefitting from its appeal to customers who want to travel but are wary of staying in large hotels [48]. As the example of Airbnb suggests, the story of the pandemic’s long-term impact on gig work may be one of disruption, rather than overall growth or decline.

While app-based gig work has ebbed and flowed, an interesting area of consideration is the shift of knowledge work to the gig economy [49]. Hasija, Padmanabhan, and Rampal note that jobs that are codifiable, can be done remotely, and contain a delay between value creation and consumption are most prone for transitioning to gig work [50]. A survey of HR leaders found that about one third of organizations are actively replacing some full-time employees with contingent workers [51]. In combination with this shift, Autor and Reynolds suggest the pandemic will accelerate the use of automation in organizational processes [52]. This is significant as it may have the potential to push displaced workers into various forms of gig work, especially if they have limited skillsets.

With USA unemployment rates reaching Depression-era levels at the outset of the pandemic, many have turned to gig work to supplement lost wages [46]. This influx, combined with low demand for some types of gig work, led to high competition amongst workers, threatening an already precarious sector of employees [46]. The influx of new, inexperienced gig workers and greater competition for gigs may serve to exacerbate pre-pandemic findings regarding the increased occupational safety and health risks associated with gig work [44]. At the same time, the pandemic has only served to augment those risks. Many employed in gig work (e.g., delivery workers, rideshare drivers, childcare providers) must interact with a variety of people, increasing their risk of infection [53,54,55,56]. These issues have led to articles asking whether it’s ethical “to hire someone to assume a risk you don’t want to” [57]. On top of the risk of infection, many gig workers are also subject to mistreatment and abuse from customers, which popular press articles suggest has increased during the pandemic [58,59]. Few gig workers have access to benefits typically associated with traditional employment (i.e., healthcare, retirement), creating a situation where greater risk is coupled with fewer resources [60].

### 2.3. Displacement of Work

#### 2.3.1. Pre-Pandemic Trends

Worker displacement refers to “the involuntary, permanent termination of long-term employees” [61] (p. 1). Speculation on the future of work has long anticipated widespread worker displacement, often as the result of advances in technology and automation [62,63,64]. Unemployment rates provide a useful metric of pre-pandemic displacement trends. In the USA, civilian unemployment had been steadily declining for more than a decade before the start of the pandemic. From a high of 10.0% in October 2009, the unemployment rate had fallen to 3.5% by January 2020 [65]. Notably, men (3.1%) and women (3.2%) experienced similar levels of unemployment in January 2020 [64].

While the unemployment rate captures only “jobless persons who are available to take a job and have actively sought work in the past four weeks” [66], civilian labor force participation includes employed and unemployed people as a percentage of the civilian noninstitutional population [66]. In the decade prior to the pandemic, labor force participation had declined marginally from 64.8% in January of 2010 to 63.4% in January of 2020 [67]. Additionally, the labor force participation rates of men (69.3%) and women (57.8%) in January 2020 had converged slightly, when compared to men’s (71.2%) and women’s (58.8%) rates in 2010 [67]. Further, underemployment, (i.e., those working fewer hours than they would like as well as those who are unemployed but not included in the official unemployment rate due to lack of job search activity) decreased from 5.1% in 2015 to 3.5% in 2019 [68,69,70,71]. All in all, these trends reflect a slow, yet steady, recovery from the 2008 financial crisis, with little evidence of the technology-induced displacement forecasted for the twenty-first century [63].

#### 2.3.2. Pandemic Trends

Worker displacement caused by the pandemic has far-reaching implications for individuals, organizations, and entire industries. March 2020 marked a massive loss of employment in the United States. Leisure and hospitality was one of the hardest-hit industries, losing about 459,000 jobs [72]. An additional 43,000 jobs were lost in healthcare and 46,000 in retail [72]. Overall, industries women tend to work in have been more severely affected [73].

The outset of the pandemic had a substantial impact on employment, with the civilian unemployment rate hitting 14.8% in April 2020 [65]. Of note, unemployment rates varied noticeably amongst groups. Whereas men’s unemployment was 13.1% in April 2020, women’s was 15.5% [65]. In fact, over the course of 2020, women lost 1,000,000 more jobs than men, prompting widespread concerns about gender equality [74,75,76]. Similarly, Caucasian (14.1%) and Asian (14.5%) Americans experienced markedly lower rates in April 2020 than those identifying as Black or African American (16.7%) or Latinx or Hispanic (18.9%) [64].

The cause of these disparities is difficult to pinpoint. For example, disproportionate unemployment amongst women is likely impacted by the industries dominated by women, but also by women’s greater employment in part-time and other precarious work [73,77]. Further, the greater caregiving and domestic responsibilities shouldered by women, in conjunction with widespread closings of schools and daycares, are likely contributors [78,79,80]. There is already emerging evidence of the pandemic’s disproportionate impact on parents’, and especially mothers’ work-life balance; this is discussed later in conjunction with the organization of work.

As the pandemic has progressed, unemployment and underemployment have begun to improve. The unemployment rate fell to 5.4% by July 2021 [65]. Averaging the third quarter of 2020 through the second of 2021, underemployment had fallen to 5.0% [70]. Additionally, women have gained ground. By July 2021, women’s unemployment rate (5.0%) had fallen below men’s (5.4%) [65]. Black (8.2%) and Hispanic (6.6%) unemployment continued to lag behind that of white (4.8%) and Asian (5.3%) Americans [65]. Labor force participation in July 2021 was 61.7%; the gap between men’s (67.6%) and women’s (56.2%) participation in July 2021 was essentially equal to what it had been in January 2020 [67].

These figures provide some reason for hope. Nonetheless, research suggests unemployment has long term impacts on earnings and homeownership [81], particularly when unemployment occurs during a recession [82]. In addition, the impacts on food insecurity are severe; Feeding America has projected that 1 in 8 Americans will experience food insecurity at some point in 2021 [83], with important implications for health and well-being [84]. Moreover, individuals’ abilities to cope with financial loss is not equal, and workers of color are overrepresented in the low wage workforce. Despite statistics promising economic recovery, the long-term effects of worker displacement, especially displacement of women and minorities, should not be overlooked.

### 2.4. Implications

#### 2.4.1. Implications for Research

With the COVID-19 virus, and especially the delta variant, continuing to shift the plans of individuals and organizations alike [85], the ‘new normal’ of work arrangements remains unknown. With increases in remote work, research is needed to investigate the effects of remote work on individuals who generally opted into working remotely versus those who are forced to work remotely, thus providing insight into the role of choice in shaping occupational outcomes [86]. Leadership styles, behaviors, and performance management strategies may all require adjustment, and research is needed to understand how leadership of fully remote or teleworking teams can promote or detract from healthy work design and worker well-being. Of particular concern are issues surrounding technostress and worker isolation [87,88], which require greater research attention. Remote work during the pandemic has been linked to fatigue and depression [87,88] while home confinement has been associated with decreased physical activity and less healthy eating [89], although research is needed to understand the extent to which this was due to the particular circumstances of remote work during the pandemic. Additionally, the proliferation of electronic performance monitoring (EPM), which involves tracking software, raises challenges for employee privacy, trust, and their consequent effects on well-being [90,91].

As workplaces adopt hybrid approaches, there are also important questions about potential inequities in training, work design, and leadership across remote and non-remote workers and their potential implications for occupational safety and health. The potential for remote work to remain a primary medium of work in the future also raises questions about occupational safety and health-related elements of people’s remote work environments, such as the ergonomics of at-home workstations. Improper design of computer workstations has been associated with neck and back pain, eye strain, and carpal tunnel syndrome [92], emphasizing the importance of research related to at-home ergonomics. Finally, Bapuji and colleagues remind us that the ability to work remotely is a luxury [53], especially during situations like the pandemic. Early evidence has already pointed to the disparate respiratory health impacts suffered by remote and non-remote workers during the pandemic [93]. It will be important to examine the extent to which, and ways by which, remote work during and after the pandemic contributes to health inequities tied to work.

Likewise, there are a number of implications for researchers to consider in examining the gig economy. First and foremost, researchers must determine how to systematically operationalize gig work as there are a number of issues surrounding its current measurement [40]. Second, research is needed to understand how gig work has changed or not changed in terms of its precarious elements and associated short- and long-term well-being outcomes. Gig work during the pandemic has increased in the degree of precarity at work due to the risk of virus exposure and potential increases in customer mistreatment [94]. Research is needed to investigate the policies and practices most effective at limiting the risks faced by gig workers both in terms of the availability of work and the quality, conditions, and experiences on the job along with the availability of worker benefits.

The widespread displacement of work also presents meaningful implications for researchers moving forward. In contrast to proclamations that technology may cause widespread job loss related to the future of work, an important question in light of the pandemic is the extent to which technology (e.g., virtual work) can facilitate a return to work. There are also concerns about the consequences of labor shortages in in-person work for those working in understaffed organizations. For example, hospitality organizations have struggled to hire back employees laid off at the start of the pandemic [95]. These labor shortages, common in many low-wage occupations, may have serious health and well-being implications for employees forced to cover additional tasks [96,97,98]. For example, research has detailed a variety of adverse health impacts associated with overwork (e.g., cerebrovascular/cardiovascular diseases, mental disorders) [99]. Conversely, this trend may usher in improvements in wages and benefits intended to entice back employees [11].

Additionally, while past research has demonstrated the negative long-term effects of recession unemployment on earnings [82], the pandemic presents an opportunity to focus on the long-term health and well-being effects associated with unemployment and temporary work loss (e.g., furloughs). A focus on inequity associated with race and gender could further enhance this stream of research. As jobs in specific industries have been disproportionately displaced, workers still employed in these industries may grow wary of the future of their roles, potentially leading to increased psychological strain [100]. The transboundary nature of the pandemic makes it difficult to predict how various industries may be affected in the future, potentially contributing to uncertainty for wider groups of workers, with implications for workers’ sense of security. Additionally, although unemployment seems to be recovering, research will need to examine the quality of jobs to which previously unemployed people have returned and the potential for health inequities due to disparate opportunities for higher quality jobs.

#### 2.4.2. Implications for Practice

Organizations, too, have much to consider moving forward, given the impacts of the pandemic on work arrangements. Assuming more and more individuals want to move to full-time remote work or hybrid schedules, organizations will need to make informed decisions about the advantages and disadvantages of remote work in their industry. Occupational safety and health professionals should play a valuable role in designing and implementing trainings, guidelines, and support materials regarding topics that have been identified as critical for remote workers’ well-being (e.g., ergonomics in the home office, work-life balance, effective virtual communication) to ease the transition to remote work [101].

Organizational leadership will need to ensure that remote employees, many of whom may be women and mothers, are not overlooked for deserved career opportunities [24]. Considering the health concerns associated with remote work and home confinement [87,88,89], leaders will also want to consider how they treat newly remote employees and engage in behaviors that will promote employee well-being. Authors have made a variety of recommendations for leaders to support their newly remote teams, such as emphasizing psychological safety and facilitating interpersonal connection [27]. Leaders are also encouraged to provide frequent feedback to remote teams [14,25]. Feitosa and Salas highlight some of the idiosyncrasies between the conventional virtual team’s literature and the current situation [27]. For example, in discussing trust, the authors place an emphasis on trust maintenance [27], rather than trust formation as these teams have presumably established trust prior to the pandemic.

Additionally, as organizations increasingly rely on contingent or gig workers during the pandemic and beyond, consideration needs to be given to the occupational safety and health implications of transitioning to a contingent worker model. Previous research has suggested that some types of gig work (e.g., ridesharing) present novel safety risks to workers and non-standard work arrangements are generally associated with greater workplace vulnerabilities than standard arrangements [44,102,103]. Simultaneously, many gig workers receive little to no safety training [8,103]. Further, evidence suggests that job insecurity interacts with contingent status to predict particularly negative safety outcomes [104]. Training and work design are critical first steps in addressing the safety disparities between gig workers and those employed in standard work arrangements [8]. Further, organizations considering the integration of contingent employees into their workforce should thoroughly evaluate the occupational safety and health implications both for contingent workers and for the rest of their workforce. This is critical as research has highlighted that companies tend to assume less responsibility for contingent workers compared to more permanent workers [53] and that the greater usage of contingent workers can promote job insecurity among those with more traditional contracts [105]. Overall, companies should design work in a way that encourages safe working behavior, rather than overemphasizing efficiency [103]. Finally, isolation and lack of social support pose well-being risks for gig workers and other remote workers [20,21,106]. Organizations should foster communication amongst these employees and, where possible, decrease perceptions of competition amongst them [106]. There is also a need for action at the policy level to protect workers in non-standard work arrangements; several such policies (e.g., the Portable Benefits Bill) have been proposed.

The displacement of work also has important implications for practice. The pandemic, and associated school and daycare closures have brought issues surrounding childcare and the gendered nature of domestic work to the forefront [107,108]. In order to hire, retain, and support parents, and particularly mothers, the importance of childcare has never been clearer. Additionally, the severe impacts of unemployment and underemployment make a strong case for an extended social safety net [81,82,83,84,109,110]. This argument is further strengthened by ‘pandemic success stories’, like New Zealand [111,112].

Further, increased job displacement during a global pandemic can trigger employee fears that the stability of their industries and their own health are at risk, potentially leading to a host of negative well-being outcomes. Organizations must be mindful of such fears as they ask employees to perform their work in new and different ways. Communication and support are paramount [113]. Additionally, policymakers may want to explore mechanisms to provide support to workers facing unemployment or pathogen exposure.

## 3. Compensation and Benefits

Closely related to questions about what work will look like in the future are questions about the quality and conditions of work as well as issues of wage stagnation and income inequality [114,115]. There are concerns about a “race to the bottom” where the combination of precarious employment systems and poor social safety net mechanisms in the United States leave many without necessary benefits to ensure health and safety [116]. With this backdrop, the following sections provide an overview of the impact of the pandemic on issues pertaining to employee compensation (i.e., wage growth and the wage inequality gap) as well as worker benefits and policies, particularly access to paid sick leave, paid family leave, and hazard pay. Paid sick leave is defined as the right to take time off due to an illness while still receiving compensation. Paid family leave refers to the right to get compensated when taking time off to care for a sick family member, while hazard pay entails getting paid extra for performing difficult or dangerous work [117,118].

### 3.1. Compensation

#### 3.1.1. Pre-Pandemic Trends

Lack of employee wage growth has been highlighted by NIOSH as integral to occupational safety and health issues and has been identified as a significant problem for workers in recent literature [119,120,121]. Prior to the pandemic, the Federal Reserve Bank of Atlanta noted that we had yet to reach pre-2008 financial crisis wage growth levels [122], which peaked at approximately 4.5% in mid-2007, fell as low as 1.7% in early 2010, and was 3.5% prior to the pandemic (late 2019–early 2020). This is critical to note as it is documented that wage growth around 4% is necessary for employees to actualize the benefits of macro-economic growth, such as increased disposable income [123]. Under this threshold, it becomes difficult for employees to regain the wage resources necessary to help them bounce back from financial challenges experienced during a recession [124]. This lack of financial realization is detrimental when considering the host of negative well-being effects associated with constant worry over financial security and debt [125,126]. This objective data aligns with employee attitudes regarding pay as Gallup noted 40% of Americans reported feeling underpaid, highlighting pre-pandemic concerns regarding pay inequity [127].

The pre-pandemic lack of wage growth has also been coupled with rising executive pay which now dwarfs employee pay by a 320:1 ratio [128]. This ratio needs to be considered in conjunction with previous research, which has highlighted the negative effects associated with rising wage inequality on a number of employee well-being outcomes [129]. Specifically, rising wage inequality has been conceptualized as a chronic stressor, creating a condition in which employees perceive limited access to the resources needed to cope with this stressor, thus leading to maladaptive coping and straining effects [129]. At a macro level, income inequality has been shown to have negative implications for outcomes, such as population health and health inequalities [130]. Taken together, this evidence suggests employees have been feeling the dual brunt of growing wage inequality and stagnation even prior to the current pandemic.

#### 3.1.2. Pandemic Trends

The economic precarity brought on by the pandemic only serves to exacerbate the negative implications associated with wage inequality and stagnation. Two types of evidence provide indications of how the pandemic is shaping trends in wages: overall change in wage growth and new job offer wage growth. New job offer wage growth refers to pay increases received when obtaining a new job. This allows us to look at the broader wage stagnation landscape while also discussing nuanced data regarding how wages are being distributed for new jobs.

In a whitepaper published by PayScale [131], researchers note that yearly percentage wage growth decreased from 1.2% growth between July 2018 and July 2019 down to 0.3% growth between July 2019 and July 2020. This highlights the rapidly declining trend of employee wage growth during the pandemic, and its disparate impact on industries that typically employ more low wage, minority, and female workers. When examining this data at an industry level, the hardest-hit industry was retail and customer service which experienced a 4.7% decline in yearly percentage wage growth between July 2019 and 2020 while the technology industry experienced a wage growth increase of 3% in the same time period. Overall, women have only experienced a 0.2% increase in yearly percentage wage growth compared to men’s 1.2% wage growth [131]. Although overall year-over-year wage growth levels have still remained somewhat stagnant between 2020–2021 (approximately 0.5% wage growth), there is some evidence that specific industries that were hardest-hit at the onset of the pandemic are beginning to rebound (e.g., food service and retail; approximately 4% wage growth between 2020 and 2021) [132].

When examining these trends in relation to new job offer wage growth, we find a greater disparity exists. PayScale researchers note a decline in new job offer wage growth from 3.3% between July 2018 and July 2019 down to 0.8% between July 2019 and July 2020. With regard to industry trends, we find similar results with retail and customer service employees experiencing a 7.8% decline in new job offer wage growth between 2019 and 2020 while the non-profit and technology industries continue to experience positive new job offer wage growth of 4.2% and 2.8% respectively [131]. Said otherwise, employees in customer service industries experienced an 8% drop in wage growth when accepting a new job in 2020 as compared to 2019. When considering rehire pay during the pandemic, which is defined as pay received by employees who are rehired by their organizations, a recent survey of USA small businesses reports that employee headcount levels are fairly close to pre-pandemic levels while hour and pay rate reductions are currently trending upward [133]. Taken together, these reports paint a grim picture regarding the state of employee wage growth during the pandemic, serving to further exacerbate the negative well-being and public health outcomes associated with rising wage inequality.

### 3.2. Employee Benefits

#### 3.2.1. Pre-Pandemic Trends

On the federal level, most public and private sector employees in companies with at least 50 workers are covered by Title II of the Family and Medical Leave Act (FMLA) of 1993. Under this act, employees are eligible for up to 12 workweeks of unpaid, job-protected sick or family leave during any 12-month period [134]. As of 2012, an estimated 59% of employees in the USA were eligible for protection under FMLA [135]. Government policy on hazard pay was limited to Title 5 of the Code of Federal Regulations [136], which requires federal employees to receive extra compensation for performing dangerous duties or duties involving physical hardship. Some of the hazardous duties covered include working with virulent biologicals and toxic chemical materials [136]. However, whether or not one is eligible for hazard pay is decided by the employer on a case-by-case basis, and an employee is not likely to be eligible if the duties they are performing were part of the original job description [137].

At the industry and organizational level, paid sick and family leave policies varied significantly across industries and individual companies. According to the Bureau of Labor Statistics while around 90% of private sector employees in management, business, and finance occupations (e.g., corporate executives, software engineers, bankers, and lawyers) had access to paid sick leave, only 58% of service workers did, along with 56% of workers in construction, extraction, farming, fishing, and forestry industries [138]. With regard to family leave benefits, pre-pandemic trends indicated approximately 89% of civilian workers had access to unpaid family leave, but only 17% had access to paid family leave [139]. This figure represents an increase from 2017, when only 15% of USA workers had access to paid family leave [139].

There has been substantial evidence that racial inequalities in access to paid sick and family leave benefits existed long before the current pandemic. Data from 2008–2016 suggests that Hispanic and African American workers consistently had lower rates of paid sick and family leave access and use than non-Hispanic whites [140]. Additionally, Hispanic women were the least likely to have access to paid sick leave amongst all employed women [141]. With regard to gender inequality in access to paid sick leave benefits, data from the 2009 and 2014 National Health Interview Surveys suggest men and women have had relatively equal access (approximately 60%) to paid sick leave [142,143]. In terms of access to paid family leave, only 9% of companies offered paid paternity leave to all of their employees [144]. Data previous to the pandemic suggests that there may be some disparities with regard to utilization of paid family leave between men and women to care for a newborn child. The National Partnership for Women and Families note that men typically have less access to paid family leave [145], less time available to them, and are subject to workplace stigmatization. These disparities are important to note given the positive effect that paid family leave has on families’ economic security, especially within the context of a transboundary crisis that has both economic and health implications.

#### 3.2.2. Pandemic Trends

The federal government’s response to the pandemic included the Families First Coronavirus Response Act (FFCRA), which was signed into law on 18 March 2020 and expired on 31 December 2020 [146]. It required employers (both public and private) with fewer than 500 workers to provide 80 h of paid sick leave for employees who were quarantined or experiencing COVID-19 symptoms. It also allowed for up to 10 additional weeks of paid family and medical leave at two-thirds of the regular rate of pay to care for a child, but only for workers who had been employed for at least 30 calendar days. Furthermore, in an effort to increase economic protections for individuals who lost their jobs during the pandemic, unemployment benefits were increased and extended providing individuals with up to an additional $600 per week up until the end of 2020, and $300 per week from the beginning of 2021 up until 14 March of 2021 [147]. Unfortunately, these provisions are no longer in place since they were essential for offsetting some of the economic stress induced by the pandemic.

A number of states (e.g., New York, California) have counteracted some of the economic and health issues faced by employees by enacting emergency legislation extending sick leave paid benefits and guaranteeing job protection [148,149]. These are critical benefits, especially for employees who have limited access to resources to provide care for family. Another example of state-provided benefits is the establishment of front-line employee hazard pay, which provides additional hazard pay for jobs that may not have been traditionally considered high-risk but are increasingly risky due to COVID-19 exposure (e.g., Vermont) [150].

At the organizational level, in March 2020, 31% of 301 listed companies reported granting additional paid sick leave to employees in light of the pandemic [151]. The extent to which these companies plan to maintain such policies after the pandemic subsides remains unclear. A promising sign, however, is the fact that some influential investors have been pressuring large corporations to place a greater emphasis on employee health than they had previously, and to better prepare for future health crises [152]. As Morgan Stanley analysts remarked, “Post-COVID-19, we expect a higher bar in terms of benefits offered to USA employees …” [152]. In addition to increases in paid sick leave, some companies are providing hazard pay to employees who would not receive such pay under normal circumstances. For instance, Starbucks employees who worked between 21 March and 31 May 2020 received an extra $3 per hour as part of “Starbucks Service Pay”—a form of hazard pay [153]. There have been calls at the federal level for increased hazard pay for frontline employees (e.g., food services and healthcare workers) [154].

There is limited data to draw from in order to conclude whether pre-pandemic trends of racial inequality in benefit access have shifted as a result of the pandemic. However, there may be some reason for concern. In particular, according to a nationwide survey by the National Employment Law Project in May 2020 [155], African American employees were twice as likely as white workers to have experienced or witnessed retaliation from their employer for raising health and safety concerns related to COVID-19 at their workplace. The survey included open-ended responses, in which some participants revealed that anyone taking a leave of absence may be fired. If such experiences are prevalent, this could signal that the pandemic is maintaining or even exacerbating racial disparities in access to paid sick leave benefits. On the other hand, given the expansion in paid leave benefits from the government and some private companies, there may be reason to believe that overall racial inequalities in access to these benefits may decrease post-pandemic. With regard to gender inequalities, recent evidence suggests that the pandemic has disproportionately affected women, many of whom were forced to reduce their work hours or quit work altogether to manage childcare responsibilities [156]. We conclude this is partly driven by unequal access to and use of childcare family leave, reflecting traditional societal expectations of women.

### 3.3. Implications

#### 3.3.1. Implications for Research

Occupational safety and health researchers have highlighted pay inequality as a grand challenge for future research and demonstrated its link to negative well-being outcomes [129,157]. Research on strategies to support employee wage growth and mitigate inequality would be beneficial in reducing economic stress of employees and associated negative health outcomes [124]. Similarly, a better understanding of the nuances surrounding income redistribution policies is necessary to help employees financially cope and rebound from the burdens placed on them during the pandemic [158,159].

Future research should carefully track how employee benefits and policies continue to evolve over the course of the pandemic and beyond. Pre-pandemic research has highlighted increases in employee novel perks (e.g., on-site dining, health facilities) in addition to traditional benefits at the organizational level [160]. These additional perks may become increasingly important over time as employees look to their organizations to provide a comprehensive set of perks and benefits to meet their diverse well-being needs. More research is needed to understand whether such perks meaningfully contribute to employee health and well-being and whether employee needs have shifted due to the pandemic [126]. Given the unique transboundary nature of the pandemic affecting both the health and economic well-being of employees, it is important to understand the benefits and policies that will have the greatest impact on employee well-being now and in the future [1].

Moreover, future research should investigate the downstream effects of disproportionate access to benefits across gender and race. As minorities and female employees make up the majority of blue-collar and service jobs, this further exacerbates deficiencies in access to benefits during the pandemic and beyond, with potentially critical effects for occupational safety and health, as well as well-being. Going further, it is critical to understand the types of benefits, policies, and resources that would improve the health and well-being of individuals in more disadvantaged and precarious work situations at the organizational, community, and federal levels [161]. Given voter support (75%) for federal policies aimed at providing increased access to paid leave [162], future research should seek to understand the best methods for increasing participation in the electoral process, as this could be an integral step in electing representatives that are motivated to form policies impacting these issues.

#### 3.3.2. Implications for Practice

Given recent advances of industry leaders (e.g., Disney, Starbucks, Ikea) raising their organization’s minimum wage, more organizations should consider proactively making these changes in an effort to foment employee wage growth. Additionally, governments, industry groups, and employers should consider increasing employee social safety benefits to help counteract lack of wage growth and threats of growing income inequality. To help counteract decreases in new offer and rehire wage growth, organizations need to consider developing long-term plans for restoring wages to pre-pandemic levels to help accelerate post-recession wage growth. Industry groups and governments can help provide businesses with guidance on restoring pre-pandemic wages over a specified time period [163]. For instance, specific recommendations are centered around using policy to restore economic power to the bottom 90% of employees as well as protecting employees’ rights for collective bargaining for better wages and working conditions, which have implications for a variety of public health outcomes.

The pandemic underlined that many Americans did not have adequate access to paid sick and family leave prior to the pandemic; despite responses to the pandemic, many still lack adequate access to leave. Providing adequate access to paid sick and family leave (as well as hazard pay, when necessary) is essential for maintaining a healthy and productive workplace and ensuring that the well-being needs of the workforce and their families are met. This includes the expansion of both traditional benefits as well as additional resources that enhance employee well-being (e.g., on-site health services). Given that such policies may prove costly for small businesses, government subsidies would be beneficial in allowing smaller companies to provide these benefits to their employees. Thus, it would be prudent for both government and private organizations to rethink their pre-pandemic benefit policies and consider expanding them beyond the pandemic.

Given the potential long delay in passing legislation, it would be beneficial for organizations to take an active role in providing these resources to employees in the meantime. Ensuring that all employees have adequate access to health- and well-being-related benefits is a crucial step in alleviating inequities and promoting appropriate health-related behavior for employees (e.g., staying home when sick). Organizations and industry groups should look to put forth policies targeted toward the needs of their workers, especially for segments of their workforce that tend to have limited access to employee benefits (e.g., women and minorities employed in blue-collar work and service jobs). Policies should be focused on improving safety, reducing potential for injury, especially in the context of blue-collar jobs (e.g., agriculture, construction), and promoting psychological and emotional well-being. We also encourage practitioners to challenge the use of the label “benefits” to describe health-related practices such as sick leave or parental leave. This terminology implies these employee resources are not necessary when in fact they are foundational to maintaining a healthy workforce. Given that current Department of Labor policy explicitly describes these resources as benefits, this will require sustained partnerships at the federal level to change how we think about essential health related resources, such as paid sick leave and family leave [164].

## 4. Organization of Work

Occupational safety and health and future of work initiatives highlight the role of work processes and organizational practices as important contributors to worker health and well-being, through shaping the demands faced at work and workers’ abilities to manage and flourish in their roles outside of work [7,165]. The pandemic has disrupted workplace norms and practices (e.g., social distance requirements), introduced new hazards (e.g., COVID-19 exposure), increased individual demands with short-staffing (e.g., overwork) and invaded the non-work domain (e.g., work-life conflict). These issues have been identified as contributors to stress, loneliness, and burnout, suggesting that the pandemic has negatively impacted the mental health of employees [166,167,168]. This section focuses on the impact the pandemic has had on trends related to the work-life balance and mental health of workers.

### 4.1. Work-Life Balance

#### 4.1.1. Pre-Pandemic Trends

There has been growing research interest in work-life balance as a determinant of psychological well-being. Work-life balance is defined as an “accomplishment of role-related expectations that are negotiated and shared between an individual and his or her role-related partners in the work and family domains” [169] (p. 458). Alternatively, work-life conflict reflects an incompatibility between work and non-work domains [170]. This conflict is typically studied from a ‘work interfering with family’ perspective (WIF) or from a ‘family interfering with work’ perspective (FIW) [171].

Pre-pandemic research points to differences among groups of workers in terms of work-life balance [171]. A 2005 meta-analysis found that men reported slightly more WIF whereas women have reported slightly more FIW [172]. Additionally, parents with more children report more conflict, with single parents, particularly working mothers, reporting higher levels of conflict [172]. Those with higher segmentation (i.e., keeping work and life separate) preferences rather than integration (i.e., blurring work and life boundaries) preferences were less likely to experience work-life conflict [173]. Importantly, those with less income are less likely to experience work-life balance due to the lack of bargaining power with one’s work [174,175]. Despite these individual differences, writers caution that work-life balance is a societal issue rather than one simply belonging to one worker group [176].

Organizational policies play a role in workers’ experiences of work-life balance and conflict. Relevant policies include flextime (i.e., adjusting when you work on a particular day), compressed work weeks, telework, remote work, and various forms of leave [177]. While these arrangements may not apply to all occupations (e.g., nurses largely cannot work remotely), other allowances (e.g., flexible shift scheduling for nurses) can be afforded to help employees achieve work-life balance [178]. Along with flexible practices, organizational culture can play a role in promoting work-life balance [179]. Research has suggested that, while flexibility practices are generally helpful in decreasing work-life conflict [177], inequalities exist in the accessibility of these practices. For example, although lower-income workers (e.g., retail food, hotel) benefit the most from flextime, they are also least likely to have access to flextime [180]. Overall, BLS data suggests the percentage of full-time wage and salary workers who have adjusted their start and stop times in some way had gone from 27.6% in 1997, to 57% by 2018 [181,182]. In terms of family leave, 12% of civilian workers had access to paid family leave in March 2012 and that percentage had risen to 17% in March 2018 [183,184]. In line with future of work trends [185], available data suggests organizational policies promoting work-life balance have become slightly more common over the past decade. However, even before the pandemic, there were childcare deserts, especially in lower income neighborhoods [186].

#### 4.1.2. Pandemic Trends

For many workers, the rapid spread of remote work has augmented challenges associated with blurring the lines between the work and family domains (i.e., decreased segmentation). In particular, BLS data suggests that employees in the private sector have been consistently working more hours per week on average since May 2020 compared to pre-pandemic levels [187]. This is particularly true for industries that are well-suited for remote work (e.g., finance) as compared to industries like manufacturing and hospitality [36,187]. While longer workdays may be the norm for remote workers, it is difficult to assess whether this increase is offset by the removal of commuting which has been noted as a benefit for many remote workers [188].

Meanwhile, essential workers, or workers identified as necessary to the everyday functioning of society (e.g., healthcare workers, grocery workers), have experienced increased stress as a result of overwork and have not had access to the paid leave that they need as a result of being exposed to COVID-19 or taking care of exposed loved ones at home [189]. On top of the inherent increased risk essential workers face by interacting face-to-face with people, research has found these individuals did not decrease their mobility patterns with the onset of the pandemic suggesting higher transmission risk [190]. This is likely a result of essential workers having less flexibility in meeting their responsibilities, thus forcing them to carry on their day-to-day work in spite of the increased virus-related risk [191]. Black or African American workers are more likely to be employed in essential industries, especially those with lower pay [192,193]. Many of those employed in essential occupations (e.g., home health aides, other health care support roles) also have an increased prevalence of underlying health conditions or risk factors [194], making the greater risk of work-related exposure to the COVID-19 virus particularly dangerous. As noted previously, bargaining power to establish work-life balance is lower among low-income individuals, further exacerbating threats to essential worker’s health and well-being [174,195]. To put it bluntly, there have been two realities for workers in the USA during the pandemic. In one, employees have been forced to adapt as work abruptly invaded their home. In the other, workers attempt to balance physical, mental, and financial survival as they are exposed to safety risks, suffer higher stress and burnout, and lack supportive organizational policies.

With widespread closures still impacting schools and other childcare arrangements, it is clear that the pandemic has especially impacted work-life balance for working parents. The implementation of remote learning from home during standard business hours means that working parents have had to manage both work and family roles simultaneously—or make new provisions for childcare. According to two independent studies, mothers were disproportionately impacted by the pandemic in terms of unemployment, labor force participation, and a slower recovery than other non-parent adults [196,197]. Additionally, the number of families with at least one member unemployed increased to 8.1 million in 2020 compared to 4.1 million in 2019 [198]. Specifically, women with children under 18 decreased in labor force participation, which includes both employment as well as looking for employment, from 72.3% in 2019 to 71.2% in 2020 [198]. Taken in conjunction, these findings suggest many mothers have withdrawn from the workforce to some extent, likely as a result of increasing caretaking responsibilities. As Robinson, Engelson, and Hayes [199] argued with reference to childcare issues faced by workers in the healthcare sector, “This poses incalculable risks to families, science, and society.”

### 4.2. Mental Health

#### 4.2.1. Pre-Pandemic Trends

According to data from the Substance Abuse and Mental Health Services Administration (SAMHSA), mental health issues have been increasing for several years among USA adults before the pandemic. As of 2019, approximately 20.6% of adults reported having any mental illness, reflecting a 2.9% increase since 2008 [200]. The percentage of adults with any mental illness receiving services also increased from 40.9% in 2008 to 44.8% in 2019 [200]. At the same time, however, 26% of adults with any mental illness perceived an unmet mental health service need; this is a higher percentage than every year since 2008 [200]. Substance use disorders co-occurring with mental illness among adults had also increased from 3.3% in 2015 to 3.8% in 2019 [200]. Similarly, both researchers and popular press articles have observed that fatigue and burnout are on the rise [201,202,203], leading to increased threats of workplace accidents and stress [204,205].

In a survey by the American Heart Association CEO Roundtable, 76% of workers reported having struggled with an issue which impacted their mental health in 2018, with 42% of those respondents having been diagnosed [206]. Comparing data from 2003–2012, researchers found some industries, like management and manufacturing, reported decreasing rates of substance use disorders while other industries remained largely the same [207].

Employee assistance programs (EAPs) represent one way organizations have tried to provide resources to employees afflicted with mental health disorders; however, sources indicate utilization rates were below 10% before the pandemic, bringing doubt to their value and reach [208]. Some experts have predicted further expansions into mental health benefits as part of employer health benefits plans, potentially as a result of industry trends [209]. In a survey gauging workplace health promotion in the USA, nearly half (46.1%) of the responding worksites had a workplace health promotion program in place with occupational safety programs representing the majority of workplace health-related programs [210]. Generally, small worksites (10–24 employees) were less likely to have a program in place than larger organizations [210]. This is concerning considering that, at the time, approximately 21 million people (16.4% of private sector employees) worked for businesses with fewer than 20 employees [211].

#### 4.2.2. Pandemic Trends

Emerging research points to the pandemic as a major challenge for mental health due to isolation, threats to life and livelihoods, and extreme working conditions. One study found that, whereas the prevalence of depressive symptoms among USA adults was about 8.5% before the pandemic, by April of 2020, that percentage leapt to 27.8% [212]. Low income, having less than $5000 in savings, and COVID-19 exposure were associated with greater risk of depression [212]. Additionally, the prevalence of substance use and suicidal ideation among employed individuals has been shown to have increased as a result of trauma or stress-related disorders associated with the pandemic [213]. While all workers have been impacted by the pandemic, essential workers (e.g., healthcare workers, grocers) have carried a particularly large burden [213,214,215]. Research indicates increased suicidal ideation (21.7%) and at least one adverse mental or behavioral health symptom (54.0%) among many essential workers [213,214]. It is important to note that women are overrepresented in this type of work, including in healthcare [216]. Thus, such mental health effects further contribute to the pandemic’s disproportionate impacts on women.

In a similar vein, workers are experiencing unprecedented levels of fatigue and burnout [217,218]. Among those suffering the most, healthcare workers directly dealing with COVID-19 patients are experiencing heightened physical and mental fatigue, as well as burnout [219]. Other groups of essential workers (e.g., service workers, grocery store employees) have similarly reported mental strain as well as fear and frustration over the burden they face at work due to a lack of resources and workplace and community support [58,220]. Additionally, healthcare workers are reporting increases in compassion fatigue, or an inability to care for patients largely due to the overwhelming nature of the task at hand [221]. The current surge is likely to exacerbate this further [222]. These reports suggest the potential for serious long-term mental health ramifications, particularly among essential employees.

While organizations have largely increased communication efforts in promoting EAPs in response to the pandemic, there is little evidence to suggest significant improvements to the mental health resources offered across a majority of organizations [223]. Even among public health employees, data suggests that only 11.7% used EAPs during March and April 2021 despite 66.1% having EAP availability [224]. However, telehealth appointments have risen, which may have the benefit of expanding reach and coverage of mental health and other healthcare services. One report has found telehealth visits increased 154% in the last week of March 2020, when compared to the same time period in March 2019 [225]. Overall, the pandemic has exacerbated mental health issues in the USA, necessitating greater mental health prioritization among organizations, industry associations, and policymakers alike.

### 4.3. Implications

#### 4.3.1. Implications for Research

It will remain paramount for research to monitor mental health among workers, and those forced to leave the labor force. For the foreseeable future, remote workers are subject to increased isolation while essential employees continue to face a very different reality of having to balance personal risk with their financial needs [213,214,226]. This kind of disruption has likely affected the way these workers have considered their work-life balance. Remote workers struggle to balance multiple roles (e.g., childcare and employee) with no clear boundaries [227]. Essential workers also must juggle these roles while simultaneously pitting their mental and physical health against their financial security [228,229]. Future research will need to examine the role of organizational and government policies and practices in enabling workers to cope with these challenges and attenuating the long-term negative effects on employee well-being and mental health. There is a particular need for research to examine the experiences of essential workers during and after the pandemic. This research will need to acknowledge the over-representation of racial and ethnic minorities as well as women in ‘essential’ occupations [205,230]. In general, research is needed to understand how the stressors and demands of the pandemic shape people’s health risks and health behaviors both proximally and distally. For example, poor diet and alcohol consumption as a result of the pandemic can increase cardiovascular health risk and may have other health implications as well (e.g., poor mental health) [231].

The pandemic has made mothers, in even some of the more egalitarian societies in the world, reckon with the impacts of gender norms [232]. As we hopefully enter a post-pandemic time period, research will need to investigate the long-term effects of this impact and consider if and how mothers and other caretakers who left the workforce choose to re-enter. Considering how abruptly many caretakers had to withdraw from the workforce as a result of at-home responsibilities, it is critical to examine the role flexible arrangements or other practices play in assisting re-entry into the workforce and subsequent work-life balance. Additionally, it will be worthwhile to examine the extent to which the workforce in general comes to value greater work-life balance as a result of experiences during the pandemic, as has been proclaimed by popular press reporting of the ‘Great Resignation.’ [233]. Although prognostications about the future of work have speculated for decades that technological advances would leave more time for leisure, this has not come to fruition. It may be a crisis, rather than technological capabilities, that catalyzes greater movement towards work-life balance and workplace well-being.

Research should continue analyzing the effectiveness of EAPs in organizations in an effort to understand the degree and consequences of underutilization. Additionally, research should consider what kind of alternative mental health resources can be provided by organizations to better support their workers as well as how healthy work design practices can reduce ill-being during crises. Finally, with the rise of telehealth, research is needed to understand changes that can be made to enhance its accessibility and effectiveness as a tertiary intervention strategy [234,235].

As the pandemic evolves and different workplaces and governments enact a range of different policies, research is needed to understand how these changing policies (and uncertainty around these policies) impact workplaces and worker physical and mental health. Research is needed to examine how the inconsistent enactment of health and safety policies across workplaces, states, and national governments may further enhance health inequities faced by workers. Researching national and organizational responses around the world could help provide guidance to practitioners and policymakers to make the informed and appropriate decisions for both the current pandemic and future similar events that may arise.

#### 4.3.2. Implications for Practice

Industries and organizations need to consider how their culture shapes the way employees view issues like mental health and work-life balance. Internal communications using language that encourages self-sacrifice and less segmentation, as well as organizational practices that are not equitable and understanding of different employees’ life situations will only exacerbate work-life conflict [236]. Researchers have advocated for policies that increase access to paid sick and family leave, mandate emergency backup staffing especially among essential services, and allow employees to request flexible and reasonable work hours [237]. While these requests may seem like a tall order, research originating in the UK suggests labor union participation and collective bargaining can be pivotal in achieving such policy initiatives [238].

As discussed earlier, more individuals have been able to engage in remote work than ever before. Employees and employers are now grappling with establishing where the line between work and non-work starts and ends. Flexible work practices can be one tool for workers to better manage their work-life balance. Workers unable to work remotely can still benefit from other forms of flexibility (e.g., flextime, compressed workweeks) [178], strengthening the call for policymakers and organizations to increase the availability of multiple forms of flexible work arrangements. At the same time, it is important that workers have a voice in determining their schedules and that work provides a means for financial sufficiency. Expansions in organizational and governmental policies would allow more employees, especially those disproportionately impacted by the pandemic, to carve out their own form of work-life balance.

The severity of the current landscape of mental health among workers (and those forced to leave the labor force) suggests that mental health treatment needs to extend beyond EAPs [223]. Organizations should consider playing a more active role in facilitating mental health resources for their employees like utilizing health advocates that help company employees with a variety of requests, such as help with making appointments and ensuring they are knowledgeable in how to use mental health resources available to them. Organizations have an important role to play in providing safe workplaces, resources, and support to promote employee welfare.

While recommendations might be made to help ease some of the negative impacts of the pandemic on an individual level, it is important to realize the magnitude of this event and in doing so, recognize the need for large-scale remedies, such as those provided by public policy and legislation. Greater unemployment benefits, leave policies, and access to healthcare would not only benefit employees who utilize these resources; it should also provide a safety net that could reduce the impact of job insecurity on well-being [237,239]. The upheavals associated with the pandemic have illustrated the importance for future of work preparations to consider both work-engendered trends (e.g., remote work) and disruptive events that can shape how these trends will unfold over time [240].

## 5. Conclusions

Speculation on what the future of work will look like is rampant. As summarized in Table 1, the pandemic has accelerated certain trends associated with the future of work, while shifting the course of others. Unfortunately, many of the changes induced by the pandemic appear to exacerbate work-related inequities, which have critical implications for health. Our analysis suggests there are important opportunities for researchers and practitioners to address these trends and lead the way in creating a healthier and safer future of work in America.

## Figures and Tables

**Table 1:** Summary of the pandemic’s impact on the future of work topics.

Future of Work Topic	Pandemic Trend Impact Summary
Remote Work	There is reason to expect a long-term increase in the prevalence of remote work, particularly among specific industries and demographic groups.
Gig Work	The pandemic disrupted the gig economy by shifting demand and increasing competition as more workers looked toward gig work to compensate for unemployment or underemployment.
Displacement of Work	The pandemic’s onset brought about widespread unemployment, especially amongst vulnerable populations (e.g., racial minorities and women, particularly mothers). Despite early signs of economic recovery, the long-term implications of unemployment and underemployment must not be overlooked.
Compensation	Wage inequality and wage growth trends both suggest the growing wage inequality pre-pandemic has been exacerbated during the pandemic while employee wage growth levels have been stagnant.
Benefits	The pandemic has spurred a number of federal and state policies targeted at increasing employee access to benefits (e.g., unemployment, paid leave). However, evidence suggests disproportionate racial and gender inequalities in employer-provided benefits remain.
Work-Life Balance	Overall, the pandemic has presented several different challenges for work-life balance. While some have been forced to adapt to work’s intrusion of the home space, others risk their physical, mental, and financial well-being in order to continue working in person.
Mental Health	Despite the increase in telehealth utilization, mental health concerns (either diagnosed or undiagnosed) increased, in part due to a confluence of financial, physical, and emotional strains brought on by the pandemic.

## Data Availability

Across the sections presented in this paper, publicly available data were analyzed in this study. For specific data inquiries, refer to the appropriate reference listed in the manuscript. Below is a list of the commonly used data sources: Bureau of Labor Statistics (BLS; https://www.bls.gov/), Department of Labor (DOL; https://www.dol.gov/), Substance Abuse and Mental Health Services Administration (SAMHSA; https://www.samhsa.gov/).
